# Supplementary figures and images for: Maternal metabolic status and in-vitro culture conditions during embryonic genome activation deregulate the expression of energy-related genes in the bovine 16-cells embryo
Source: PLoS One. 2023 Aug 25;18(8):e0290689. doi: 10.1371/journal.pone.0290689 (PMC10456174; doi:10.1371/journal.pone.0290689)

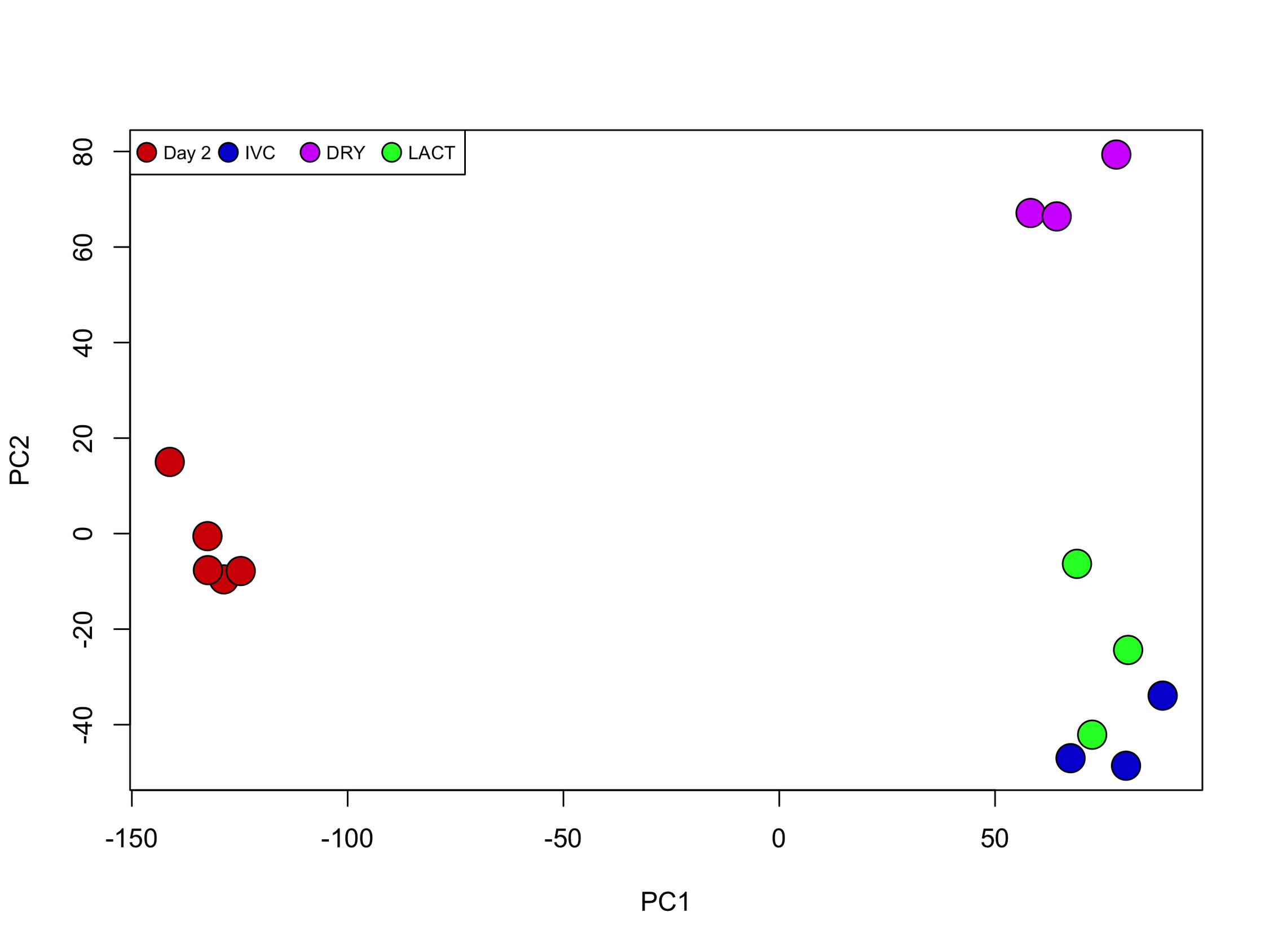

Supplement: S1 Fig — Day 4 embryos underwent embryonic genome activation during in-vitro culture (IVC) or in the oviduct of nonlactating (DRY) or lactating (LACT) dairy cows. (TIF) [file pone.0290689.s002.tif]

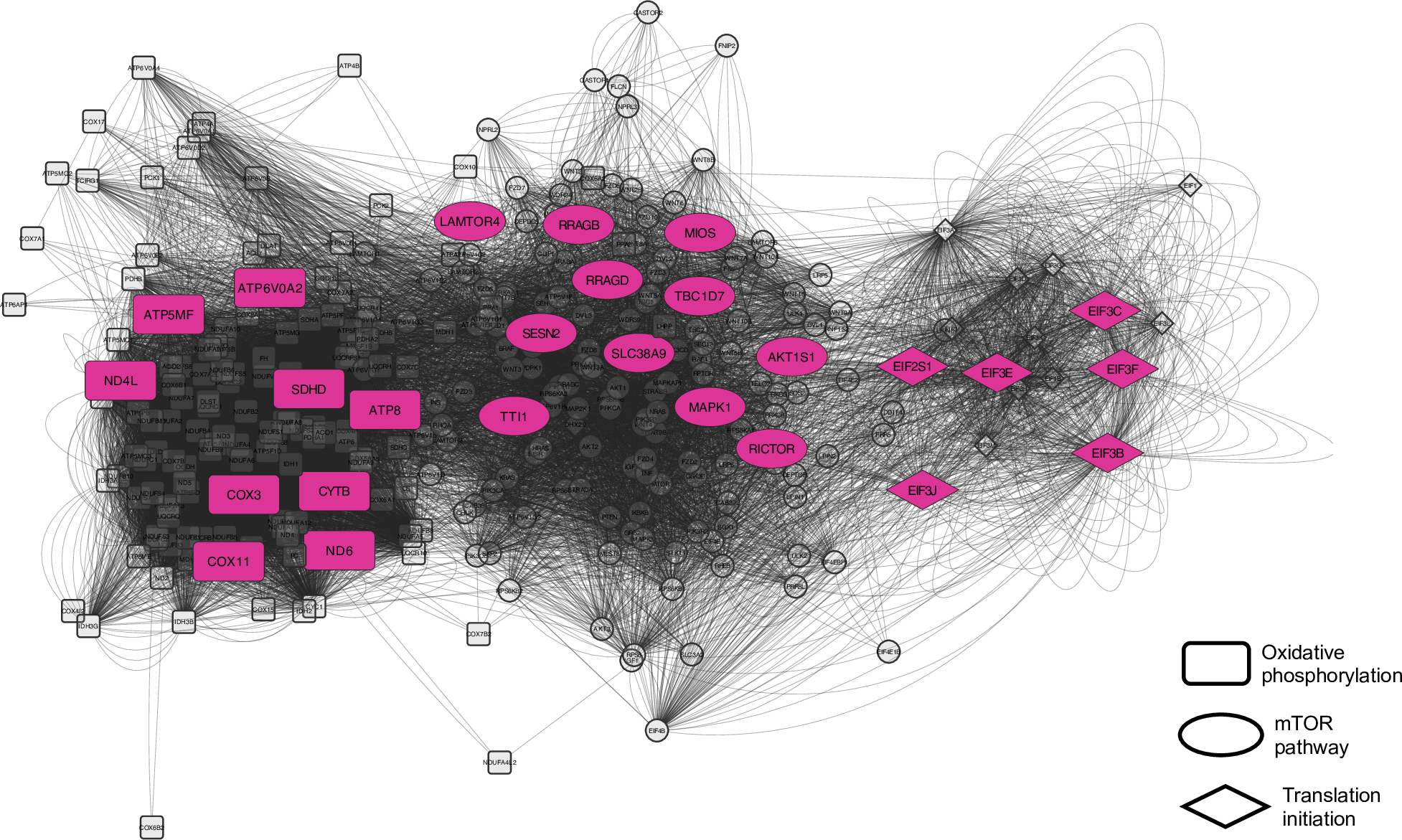

Supplement: S2 Fig — The highlighted nodes are genes differentially expressed between Day-4 embryos that underwent embryonic genome activation in different conditions. (TIF) [file pone.0290689.s003.tif]

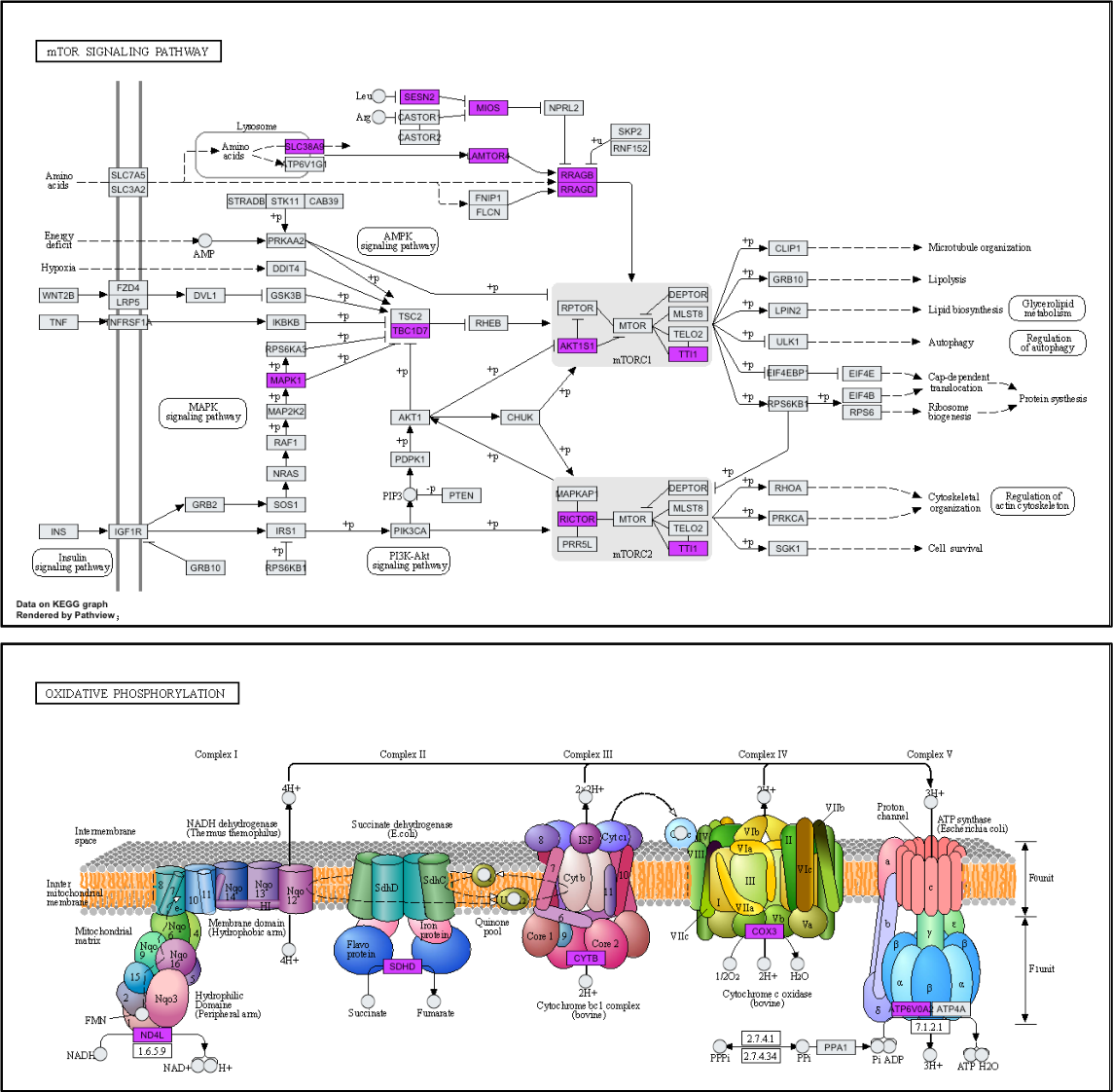

Supplement: S3 Fig — Genes in purple are differentially expressed genes between Day-4 embryos that underwent embryonic genome activation in different conditions. (TIF) [file pone.0290689.s004.tif]
