# Supplementary material for: Maternal metabolic status and in-vitro culture conditions during embryonic genome activation deregulate the expression of energy-related genes in the bovine 16-cells embryo
Source: PLoS One. 2023 Aug 25;18(8):e0290689. doi: 10.1371/journal.pone.0290689 (PMC10456174; doi:10.1371/journal.pone.0290689)
Supplement: Attachment — Submitted filename: Response to Reviewers.docx [file pone.0290689.s009.docx]

PONE-D-23-15571

==============================
ACADEMIC EDITOR:

Although both reviewers found some interesting findings, they also have major concerns. Please respond to the reviewers' questions and submit the updated version of the manuscript. Particularly, the discussion should be made more precisely based on the findings of this study.

AU: We appreciate the positive feedback and the constructive criticism of the manuscript by both reviewers. We have addressed all of the comments by providing a response below and, where appropriate, by modifying the manuscript.

==============================

Please submit your revised manuscript by Aug 21 2023 11:59PM. If you will need more time than this to complete your revisions, please reply to this message or contact the journal office at [plosone@plos.org](mailto:plosone@plos.org). When you're ready to submit your revision, log on to <https://www.editorialmanager.com/pone/> and select the 'Submissions Needing Revision' folder to locate your manuscript file.

Please include the following items when submitting your revised manuscript:

- A rebuttal letter that responds to each point raised by the academic editor and reviewer(s). You should upload this letter as a separate file labeled 'Response to Reviewers'.
- A marked-up copy of your manuscript that highlights changes made to the original version. You should upload this as a separate file labeled 'Revised Manuscript with Track Changes'.
- An unmarked version of your revised paper without tracked changes. You should upload this as a separate file labeled 'Manuscript'.

If you would like to make changes to your financial disclosure, please include your updated statement in your cover letter. Guidelines for resubmitting your figure files are available below the reviewer comments at the end of this letter.

If applicable, we recommend that you deposit your laboratory protocols in [protocols.io](http://protocols.io) to enhance the reproducibility of your results. [Protocols.io](http://Protocols.io)assigns your protocol its own identifier (DOI) so that it can be cited independently in the future. For instructions see: <https://journals.plos.org/plosone/s/submission-guidelines#loc-laboratory-protocols>. Additionally, PLOS ONE offers an option for publishing peer-reviewed Lab Protocol articles, which describe protocols hosted on [protocols.io](http://protocols.io). Read more information on sharing protocols at <https://plos.org/protocols?utm_medium=editorial-email&utm_source=authorletters&utm_campaign=protocols>.

We look forward to receiving your revised manuscript.

Kind regards,

Birendra Mishra, DVM, PhD
Academic Editor
PLOS ONE

Journal requirements:

When submitting your revision, we need you to address these additional requirements.

1. Please ensure that your manuscript meets PLOS ONE's style requirements, including those for file naming. The PLOS ONE style templates can be found at

<https://journals.plos.org/plosone/s/file?id=wjVg/PLOSOne_formatting_sample_main_body.pdf> and

<https://journals.plos.org/plosone/s/file?id=ba62/PLOSOne_formatting_sample_title_authors_affiliations.pdf>

2. PLOS requires an ORCID iD for the corresponding author in Editorial Manager on papers submitted after December 6th, 2016. Please ensure that you have an ORCID iD and that it is validated in Editorial Manager. To do this, go to ‘Update my Information’ (in the upper left-hand corner of the main menu), and click on the Fetch/Validate link next to the ORCID field. This will take you to the ORCID site and allow you to create a new iD or authenticate a pre-existing iD in Editorial Manager. Please see the following video for instructions on linking an ORCID iD to your Editorial Manager account: <https://www.youtube.com/watch?v=_xcclfuvtxQ>

3. We note that Figure 1 in your submission contain copyrighted images. All PLOS content is published under the Creative Commons Attribution License (CC BY 4.0), which means that the manuscript, images, and Supporting Information files will be freely available online, and any third party is permitted to access, download, copy, distribute, and use these materials in any way, even commercially, with proper attribution. For more information, see our copyright guidelines: <http://journals.plos.org/plosone/s/licenses-and-copyright>.

We require you to either (1) present written permission from the copyright holder to publish these figures specifically under the CC BY 4.0 license, or (2) remove the figures from your submission:

a. You may seek permission from the original copyright holder of Figure 1 to publish the content specifically under the CC BY 4.0 license.

We recommend that you contact the original copyright holder with the Content Permission Form (<http://journals.plos.org/plosone/s/file?id=7c09/content-permission-form.pdf>) and the following text:

“I request permission for the open-access journal PLOS ONE to publish XXX under the Creative Commons Attribution License (CCAL) CC BY 4.0 (<http://creativecommons.org/licenses/by/4.0/>). Please be aware that this license allows unrestricted use and distribution, even commercially, by third parties. Please reply and provide explicit written permission to publish XXX under a CC BY license and complete the attached form.”

Please upload the completed Content Permission Form or other proof of granted permissions as an "Other" file with your submission.

In the figure caption of the copyrighted figure, please include the following text: “Reprinted from [ref] under a CC BY license, with permission from [name of publisher], original copyright [original copyright year].”

b. If you are unable to obtain permission from the original copyright holder to publish these figures under the CC BY 4.0 license or if the copyright holder’s requirements are incompatible with the CC BY 4.0 license, please either i) remove the figure or ii) supply a replacement figure that complies with the CC BY 4.0 license. Please check copyright information on all replacement figures and update the figure caption with source information. If applicable, please specify in the figure caption text when a figure is similar but not identical to the original image and is therefore for illustrative purposes only.

[Note: HTML markup is below. Please do not edit.]

Reviewers' comments:

Reviewer's Responses to Questions

**Comments to the Author**

1. Is the manuscript technically sound, and do the data support the conclusions?

The manuscript must describe a technically sound piece of scientific research with data that supports the conclusions. Experiments must have been conducted rigorously, with appropriate controls, replication, and sample sizes. The conclusions must be drawn appropriately based on the data presented.

Reviewer #1: Partly

Reviewer #2: Yes

2. Has the statistical analysis been performed appropriately and rigorously?

Reviewer #1: Yes

Reviewer #2: Yes

3. Have the authors made all data underlying the findings in their manuscript fully available?

The [PLOS Data policy](http://www.plosone.org/static/policies.action#sharing) requires authors to make all data underlying the findings described in their manuscript fully available without restriction, with rare exception (please refer to the Data Availability Statement in the manuscript PDF file). The data should be provided as part of the manuscript or its supporting information, or deposited to a public repository. For example, in addition to summary statistics, the data points behind means, medians and variance measures should be available. If there are restrictions on publicly sharing data—e.g. participant privacy or use of data from a third party—those must be specified.

Reviewer #1: Yes

Reviewer #2: Yes

4. Is the manuscript presented in an intelligible fashion and written in standard English?

PLOS ONE does not copyedit accepted manuscripts, so the language in submitted articles must be clear, correct, and unambiguous. Any typographical or grammatical errors should be corrected at revision, so please note any specific errors here.

Reviewer #1: Yes

Reviewer #2: Yes

5. Review Comments to the Author

Please use the space provided to explain your answers to the questions above. You may also include additional comments for the author, including concerns about dual publication, research ethics, or publication ethics. (Please upload your review as an attachment if it exceeds 20,000 characters)

**Reviewer #1:** This study explores the impact of metabolic stress caused by milk production of dairy cows in the early embryo, in particular during the -major- embryonic genome activation (EGA). The transcriptome of embryos entirely cultured in vitro was examined in contrast with embryos fertilized in vitro that developed in the genital tract of dry and lactating cows. The analysis identified a number of (new) genes / pathways differentially expressed between all types of embryos used. The design of this study is elegant, the work is well written and easy to read.

AU: We appreciate the positive feedback and the constructive criticism of the manuscript.

The main limitations of this study are the following
- High dimensionality: in spite the correct limitative statistical tests for false discovery, authors should be cautious when drawing conclusions from this manuscript. Variability is also limited within in vivo embryos, as all them were collected from a same cow (L159-160). The above concerns must be discussed

AU: We agree with the reviewer on this point, and we have included this observation in the discussion (lines 383-388, clean manuscript). Still, the plot corresponding to a principal component analysis (a dimension reduction technique; FigS1) constructed with the whole transcriptome, which depicts clusters of samples based on their similarity, showed that samples from the same treatment group clustered close together. Thus, even though the sample size was small and variability was limited within the in vivo embryos, the observed results reflected the treatment effect for all the samples in the group.

- Limited sample numbers: The use of N=3 groups per treatment in large data experiments is discouraged, as detection of outliers is more difficult and, in the case of strong divergences between one and the other two experimental groups, discarding one group ruins the experiment.

AU: The sample size of 3 per group is considered as the minimum for an inferential analysis of data derived from RNAseq (PMID: 26813401). We agree that is not possible to discard any sample if there are differences between them. Still, we are confident that the data generated with our experiment led us to the correct biological interpretation. Each sample was composed of a pool of 5 embryos, the results are biologically sound, and, as mentioned above, the principal component analysis plot shows that samples from each treatment group together when considering the whole transcriptome. In other words, there were not strong divergences between samples in the same treatment group.

- Some inconsistencies of the Discussion section need re-thinking:
L386 onwards: the results dealing with epigenetic control (i.e., H2A, H3, and H4 and the methylation process, exhibited similar expression between IVP embryos at the 2- to 4-cell (pre-EGA) and 8 to 16-cell (post-EGA)) can be difficult to explain. The authors provide appropriate references to EGA activation (42 and 43), but the facts are not the same. In contrast, other results obtained in this study appear more consistent (L399-400)

AU: We agree with the reviewer. We have modified the paragraph to state that the transcriptomic results might indicate changes at the epigenome level, but we cannot prove it with this study (lines 396-400, clean manuscript).

L423-425: Or simply is an inconsistent result obtained from sample limitations.

AU: Perhaps. We added this possibility to the discussion (lines 427-428).

Other concerns
L39-41 (abstract): unclear sentence that should be re-rewritten.

AU: Thank you. This sentence was re-written.

L99-L112: Consider whether the text described is relevant for this study.

AU: Thank you. We believe that it is important to provide detailed information about the cows employed in this study, so the reader can have an overall idea of the experiment without needing to read the previous paper.

L-244-245: re-write as follows: Functional analysis of DEG in each cluster revealed that 1413 Day 2 genes (i.e., maternally expressed) were not expressed by Day-4 embryos (Cluster 1)…

AU: Thank you, the sentence was rewritten.

Undoubtedly the network study depicted in Suppl Fig 3 represents the actual gene relationship, but the tangled mess is scarcely useful to identify something. The explanations in text given by the authors suffice.

AU: It is true that the network is tangled. Still, it provides the idea that genes in the three pathways are strongly connected and it also represents the location of the key genes in the networks.

**Reviewer #2**: Overall, the paper entitled "Maternal Metabolic Status and In-vitro Culture Conditions During Embryonic Genome Activation Deregulate the Expression of Energy-Related Genes in the Bovine 16-Cell Embryo" by Rabaglino et al. is well-written and effectively communicates the objectives, methods, and findings of the study. The paper demonstrates a high level of clarity and coherence in its writing. The language used is precise and accessible, allowing the reader to easily understand the research concepts and findings. The methodology section is thorough and well-described, allowing the reader to understand the experimental design and procedures employed in the study, especially regarding the model that was extensively characterized in previous works. The results section presents the findings of the study in a clear and concise manner, but I recommend that the authors increase the font size of the figures as they are currently difficult to read.

AU: Thank you very much for your positive feedback and constructive criticism. The figures were modified to facilitate the reading.

My main concern lies with the discussion section of the paper. The authors propose a model to examine the impact of metabolism on the molecular characteristics of embryos. However, there seems to be insufficient attention given to this aspect. In the results section, four main classes of genes were identified: those related to epigenetic mechanisms, OXPHOS, mTOR, and translation. Although the authors acknowledge their interconnectedness and susceptibility to environmental influences, the discussion section lacks reference to how the environment might be influencing these molecular statuses.

For example, the results indicate that embryos derived from LACT exhibited lower OXPHOS activity compared to DRY embryos, even in a supposed environment with low glucose and high free fatty acids (FFA). This raises questions about how to explain this observation. Besides, why did the LACT environment lead to embryos resembling those from in-vitro culture (IVC) based on the composition of both systems? How might these molecular changes in the environment be influencing the expression of genes related to epigenetics? These are some of the points that could bring valuable information to the discussion.

Given that the authors have already extensively characterized this model, I strongly suggest utilizing all available data to help elucidate these intriguing molecular findings. By incorporating additional information from their previous research, they can provide a more comprehensive explanation for the observed molecular changes and their relationship to the environment.

AU: We appreciate this comment. We have thoroughly revised and modified the discussion to provide potential explanations to the observed results between LACT, DRY or IVP embryos (lines 438-470, clean manuscript)

6. PLOS authors have the option to publish the peer review history of their article ([what does this mean?](https://journals.plos.org/plosone/s/editorial-and-peer-review-process#loc-peer-review-history)). If published, this will include your full peer review and any attached files.


If you choose “no”, your identity will remain anonymous but your review may still be made public.


**Do you want your identity to be public for this peer review?** For information about this choice, including consent withdrawal, please see our [Privacy Policy](https://www.plos.org/privacy-policy).

Reviewer #1: No

Reviewer #2: No

[NOTE: If reviewer comments were submitted as an attachment file, they will be attached to this email and accessible via the submission site. Please log into your account, locate the manuscript record, and check for the action link "View Attachments". If this link does not appear, there are no attachment files.]

While revising your submission, please upload your figure files to the Preflight Analysis and Conversion Engine (PACE) digital diagnostic tool, <https://pacev2.apexcovantage.com/>. PACE helps ensure that figures meet PLOS requirements. To use PACE, you must first register as a user. Registration is free. Then, login and navigate to the UPLOAD tab, where you will find detailed instructions on how to use the tool. If you encounter any issues or have any questions when using PACE, please email PLOS at [figures@plos.org](mailto:figures@plos.org). Please note that Supporting Information files do not need this step.
